# A Novel Paradigm for Adult‐Onset Type 1 Diabetes: Once‐Weekly Basal Insulin and SGLT‐2 Inhibition to Harness Residual β‐Cell Function

**DOI:** 10.1002/dmrr.70247

**Published:** 2026-10-27

**Authors:** Paolo Pozzilli, Richard David Leslie

**Affiliations:** ^1^ Blizard Institute Queen Mary University of London London UK; ^2^ Università Campus Bio‐Medico Rome Italy

**Keywords:** C‐peptide, once‐weekly basal insulin, SGLT2 inhibitors, type 1 diabetes

## Abstract

Type 1 diabetes (T1D) in adults is characterised by a less aggressive disease course than in children. Most patients retain preserved C‐peptide (> 0.2 nmol/L) and some of them benefit from a protracted phase without the need for insulin. Here, we propose a hypothesis‐generating therapeutic paradigm with once‐weekly basal insulin combined with an SGLT2 inhibitor (SGLT2is) as initial therapy in T1D patients with preserved C‐peptide. Once‐weekly basal insulin, approved in Europe and now also in the US, confirm comparable HbA1c reductions to insulin degludec or glargine, albeit always alongside prandial insulin. SGLT2is, approved as adjuncts in T1D in select regions, can control prandial peaks without boluses. No trials have tested the combination of weekly insulin with SGLT2is in adult T1D patients. Nonetheless, the rationale is robust especially in adults with C‐peptide > 0·2 nmol/L. In this respect, we urge proof‐of‐concept trials of 12‐months randomised studies in adults ≥ 18 years, to evaluate this combination.

Type 1 diabetes (T1D) in adults aged 18 years and older represents the majority of cases with T1D at diagnosis, often characterised by a less aggressive disease course than in children [1, 2, 3, 4]. At presentation, most patients retain preserved C‐peptide (> 0·2 nmol/L), reflecting residual β‐cell function [3]. This cohort benefits from a protracted phase without the need for insulin, yet current basal‐bolus insulin regimens—with or without continuous glucose monitoring (CGM) and hybrid closed‐loop systems—impose a heavy treatment burden, poor adherence in up to 50% of cases, and substantial costs to health systems. Here, we propose a hypothesis‐generating therapeutic paradigm: once‐weekly basal insulin combined with an SGLT2 inhibitor (SGLT2i) as initial therapy in selected adults with T1D and preserved C‐peptide. This approach leverages endogenous insulin secretion and SGLT2i‐mediated glucosuria to simplify management, enhance compliance, and potentially preserve β‐cell mass.

Once‐weekly basal insulin, such as insulin icodec approved in Europe and the US, has transformed type 2 diabetes care by reducing injection frequency from 365 to 52 per year while maintaining glycaemic control equivalent to daily analogues. Pivotal trials in T1D (ONWARDS 6, QWINT‐5) confirm comparable HbA1c reductions to insulin degludec or glargine, albeit always alongside prandial insulin. However, since hypoglycaemia risk is a relevant patient‐related outcome, this parameter should be considered when once‐weekly basal insulin is administered to T1D patients [5]. Pharmacokinetics show stable 24‐h coverage with minimal day‐to‐day variability, addressing a key driver of basal omission—a precursor to diabetic ketoacidosis (DKA) [5]. In patients with residual *β*‐cells, weekly basal insulin could potentially suffice for fasting and overnight glucose homoeostasis, obviating daily injections.

SGLT2is, approved as adjuncts in T1D in select regions, blunt postprandial glucose excursions via urinary glucose excretion, reducing insulin requirements by 10%–20%, bodyweight by 3–5 kg and total daily dose variability [6]. In addition to dapagliflozin and empagliflozin, other SGLT2i are available in Japan (ipragliflozin) for T1D management [7]. In adults with preserved C‐peptide, this natriuretic and caloric loss might synergise with endogenous insulin secretion to control prandial peaks without boluses [8]. Pooled analyses from trials, such as DEPICT and EASE, indicate HbA1c drops of 0.4–0.5%, with benefits accruing in those with higher baseline C‐peptide [9]. Critically, SGLT2is mitigate hyperglycaemia‐driven glucotoxicity, a stressor that accelerates β‐cell exhaustion at diagnosis.

This combination offers multifaceted advantages. First, DKA risk mitigation: reliable weekly basal ensures consistent insulin tone, countering omission‐related ketogenesis, while SGLT2is avert extreme hyperglycaemia—though euglycaemic DKA remains a concern in cases with Body Mass Index < 27 kg/m^2^, necessitating ketone monitoring and sick‐day rules. Second, superior compliance: once weekly injection plus a daily tablet surpasses multiple daily injections or pump therapy, particularly appealing for active adults rejecting devices. Third, cost savings: UK and Italian health service models project 20%–30% reductions versus pumps/CGM, factoring lower insulin use and fewer clinic visits [6]. Fourth, pump‐free management democratises access in resource‐limited settings. Finally, by stabilising glucose excursions, the regimen may prolong C‐peptide secretion, mirroring preservation strategies in new‐onset T1D.

No trials have tested this exact regimen, rendering it hypothetical. Existing studies pair weekly basal with prandial insulin and SGLT2is with full basal‐bolus therapy. Nonetheless, the rationale is robust: adults with C‐peptide > 0.2 nmol/L (measured fasting or stimulated) represent ∼70% of this age group, often with slower autoimmunity (including latent autoimmune diabetes in adults) [3]. Selection criteria might include patients with no DKA at onset, receiving proper diabetes education and excluding normal body weight patients. Therapy should start with once weekly insulin, titrated to fasting glucose 70–130 mg/dL, plus low‐dose dapagliflozin (5 mg) or empagliflozin (10 mg)—doses with favourable DKA profiles in meta‐analyses—escalating per CGM metrics. Rescue boluses for hyperglycaemia > 250 mg/dL, with mandatory ketone strips.

Challenges demand caution. SGLT2i‐related DKA incidence rises 2–5‐fold in T1D, often euglycaemic, underscoring need for patient selection and training. Long‐term β‐cell effects are unproven, and prandial control might falter in high‐carbohydrate diets. Nonetheless, precision medicine targeting residual function aligns with T1D heterogeneity.

We urge proof‐of‐concept trials: 6–12‐month randomised studies in adults ≥ 18 years with C‐peptide > 0.2 nmol/L, comparing weekly basal plus SGLT2i versus standard basal‐bolus. Primary endpoints include: stimulated C‐peptide, time‐in‐range (> 70%), DKA events and quality‐of‐life scores (e.g., PAID scale). Secondary endpoints include: costs, insulin dose and weight. The use of SGLT‐2i in overweight/obese patients with T1D and cardiovascular complications remains an open issue. Although the cardiovascular and renal benefits of SGLT2 inhibition are well established in T2D, these findings cannot currently be extrapolated to T1D in which the potential benefits must be weighed against the substantially increased risk of diabetic ketoacidosis. Prospective studies are therefore needed to determine whether carefully selected, high‐cardiovascular‐risk patients with T1D may derive a net clinical benefit from SGLT2 inhibition.

## Author Contributions

**Paolo Pozzilli, David Leslie:** conceptualization (equal), writing – original draft (equal), review and editing (equal), approval of the final version submitted for the publication (equal).

## Funding

The authors have nothing to report.

## Conflicts of Interest

The authors declare no conflicts of interest.

## Data Availability

Data sharing not applicable to this article as no datasets were generated or analysed during the current study.
